# Applying tiling and pattern theory in the design of hollow-core photonic crystal fibers for multi-wavelength beam guidance

**DOI:** 10.1038/s41598-020-76747-2

**Published:** 2020-11-12

**Authors:** Zev Montz, Amiel A. Ishaaya

**Affiliations:** School of Electrical and Computer Engineering, Ben-Gurion University of the Negev, Beer Sheva, 8410501 Israel

**Keywords:** Fibre optics and optical communications, High-harmonic generation

## Abstract

We apply tiling and pattern theory in the design of hollow-core photonic crystal fibers for guiding light in multiple spectral bandgaps. By combining two different glass apexes in a single [3^6^;3^2^.4.3.4] 2-uniform tiling, transmission regions with fundamental, second and third harmonic wavelengths are supported. This cladding design may also be an excellent candidate for high power beam delivery of Er/Yb, Nd:YAG and Ti:Sapphire laser sources.

For the past two decades, hollow-core photonic crystal fibers (HC-PCFs)^1,2^ have had a tremendous impact on nonlinear optics. HC-PCFs have allowed to reduce the threshold of many nonlinear processes by several orders of magnitude^3^. This dramatic threshold reduction was obtained by tight confinement of the fundamental mode (FM), and by increasing dramatically the light-gas interaction length. HC-PCFs that guide a FM within a certain range of frequencies and reject frequencies outside this range are termed hollow-core photonic bandgap fibers (HC-PBGFs). It was shown that the FM transmission loss within the bandgap is limited by scattering losses at the core boundary^4^. Many nonlinear processes, such as high harmonic generation, require multiple spectral bandgaps that will guide the laser pump signal and generated harmonics with low transmission losses. It was shown that PBGFs with interstitial holes^5^ and high air-filling structures^6–9^ can guide light in two separate bandgaps. Yet, the ratio between their central normalized frequencies at the air line $ck_z/w=1$ is not suitable for second harmonic (SH) and third harmonic (TH) guidance. Recently it was theoretically demonstrated that HC-PBGFs can guide light in two well separated bandgaps suitable for TH guidance^10^. Such cladding designs can guide the FM and TH Gaussian modes with low transmission losses; yet, it is unclear if phase-matching of these modes is feasible.

Several approaches have been proposed to phase-match second harmonic generation (SHG) and third harmonic generation (THG) in PCFs. In solid core PCFs, phase-matching a Gaussian total internal reflection mode with a Gaussian bandgap mode was theoretically proposed^11^ and experimentally demonstrated^12^. In HC-PCFs, it was theoretically proposed to quasi phase-match high harmonic generation by modulating the phase of ionization electrons using a counter-propagating beam^13,14^. THG was experimentally demonstrated in an Ar-filled kagome fiber by counterbalancing the fiber dispersion and the gas dispersion with two different order modes^15^. SHG was experimentally demonstrated in a Xe-filled kagome fiber by applying an external DC field^16^. Kagome fibers^3^ are an excellent platform for high harmonic generation since their transmission region is broadband.

Another class of hollow-core fiber that is attracting much interest are antiresonant fibers^17^. Recently it has been demonstrated that antiresonant fibers can guide light with very low transmission losses^18^. Obtaining such low transmission losses in a hollow-core fiber is quite remarkable; yet, it was demonstrated with a relatively large core diameter, namely ${\sim 37}\, {\upmu \hbox {m}}$. Large core diameters are very common in antiresonant fibers since the antiresonant reflecting optical waveguide (ARROW) model, which is used for designing antiresonant fibers, breaks down at small core diameters^19^. Small core diameters are preferable for enhancing high harmonic generation, and therefore it is necessary to investigate new approaches for designing HC-PCFs with small core diameters and low transmission losses at the FM and its higher harmonics^10^. Such HC-PCFs could withstand high energy pulses that cannot be guided in solid core PCFs.

Here we show for the first time, to the best of our knowledge, how to apply tiling and pattern theory^20^ in the cladding design of high air-filling HC-PCFs and guide light in multiple spectral bandgaps. With this approach it is possible to design cladding structures that can guide a FM, SH and TH simultaneously. All of the modes, which are guided in separate bandgaps, have Gaussian distributions; therefore, their spatial overlap is exceptionally good.

## Results and discussion

**Figure 1:**
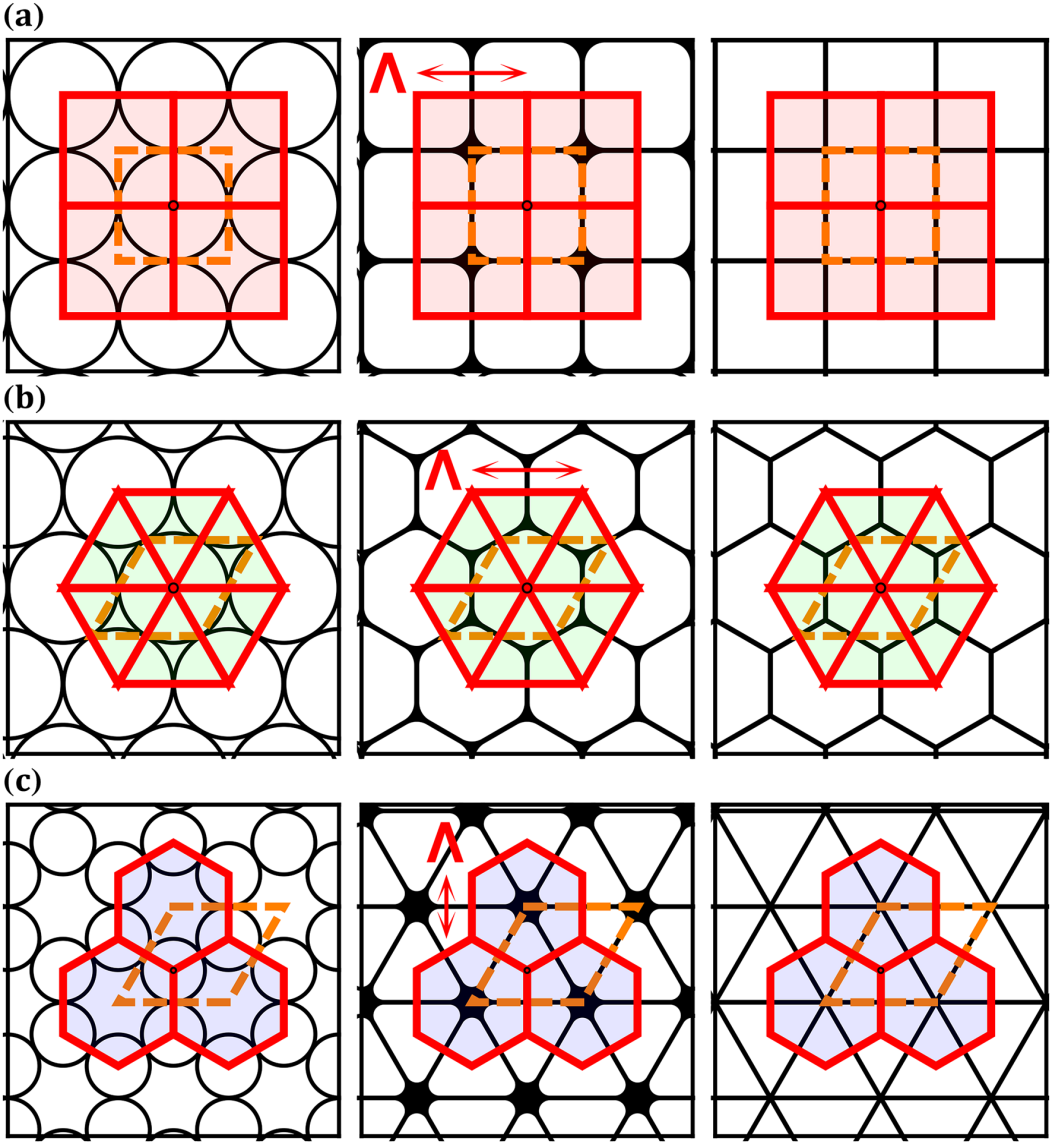
(**a**) Square, (**b**) triangular and (**c**) hexagonal uniform tilings. In all columns, the tilings (polygons with red edges) surrounding each vertex and the unit cells (polygons with orange dashed edges) of the structures are shown. Left column shows the preforms of the high air-filling structures, center column shows the high air-filling structures after pressurizing the thin capillaries during the fiber draw and right column shows the Laves tilings.

Pressurizing preform thin glass capillaries, which are centered on the vertexes of uniform tilings (Fig. 1, left column), creates high air-filling structures with apexes that are located at the center of the tilings and glass struts that are perpendicular to the tilings’ edges (Fig. 1, center column). When the apex curvatures of high air-filling structures are equal to zero, we simply obtain the Laves tilings (Fig. 1, right column). Throughout the manuscript, apex curvatures are identical to the definitions in^21^ and structures are termed according to their tilings. Apex separation in the triangular, square and hexagonal structures are ${\Lambda /\sqrt{3}}$, ${\Lambda }$ and ${\sqrt{3}\Lambda }$, respectively.  ${\Lambda }$ is the distance between the center of adjacent capillaries, which is equal to the tilings’ edges, and also equal to the square and triangular unit cell edge length (Fig. 1a,b, center column).

Bandgap formation in HC-PCFs is associated with three different resonators in the high air-filling structure: (a) apexes, (b) struts and (c) air holes^22^. The triangular (Fig. 1b) fundamental bandgap lowest frequency is bound by the silica apexes and the fundamental bandgap highest frequency is bound by the silica struts. Within the fundamental bandgap there is also a specific frequency and effective index range that is associated with the air holes. Similarly, the square (Fig. 1a) fundamental bandgap is also bound by the apex and strut resonators^23^. Interestingly, in higher order bandgaps it was shown that the apex and strut resonators can interchange their bandgap bounding positions^24^. Resonators bound the HC-PCFs’ bandgap; yet, the fundamental bandgap bandwidth originates from the properties of the single apex in the high air-filling structure^25^. The struts connecting the apexes reduce the fundamental bandgap bandwidth by bounding the bandgaps’ highest frequency; thus the struts have a similar effect as a low-pass filter. Even though most of the high frequency range is blocked by the struts, there are situations where they can support higher order bandgaps^6,8,9^. The most common way to calculate accurately the frequency bandwidth of these higher order bandgaps is with full-vectorial frequency domain methods, such as the plane wave expansion method (PWE)^26^.

**Figure 2:**
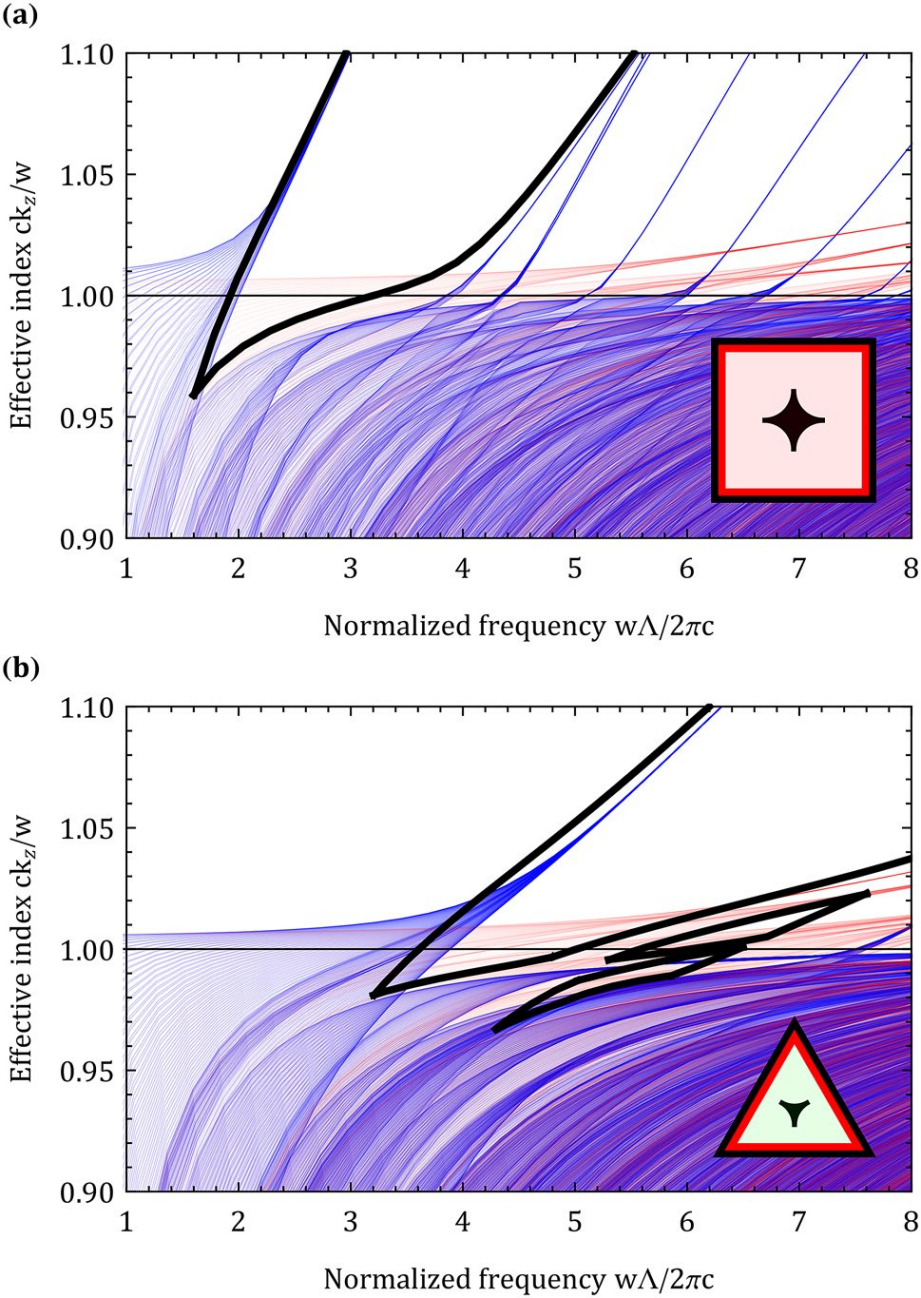
Separate band diagrams of the apexes (blue) and struts (red) in the (**a**) square and (**b**) triangular structures with an ${0.15\Lambda }$ apex curvature and ${0.01\Lambda }$ strut thickness. Bandgaps of the structures are shown in bold (black), horizontal line (black) is the air line. Figure inset shows the structures’ apexes, which are located at the center of the tilings.

Figure 2 shows the struts and apexes band diagrams of the square and triangular structures. Glass dielectric constant was set to 2.1, apex radius curvature and strut thickness were set to ${0.15\Lambda }$ and ${0.01\Lambda }$, respectively. Fixing the dielectric constant is useful since it allows to find the band diagrams in a scalable system and scale the structure according to the desired nonlinear process of interest^27^. Fused silica low dispersion allows to preserve quite well the ratio between the central frequencies of the bandgaps during the scaling process. The structures’ fundamental bandgap lowest normalized frequency is located near the apexes’ fundamental bandgap lowest normalized frequency. The struts’ band diagrams span the entire apexes’ fundamental bandgap and are known to reduce the frequency bandwidth of the structures’ bandgaps^8^. In the square structure, the struts slightly narrow the apex fundamental bandgap (Fig. 2a); yet, in the triangular structure (Fig. 2b) the struts causes the apex fundamental bandgap to be discontinuous with several bandgaps. In the triangular structure, only the first higher order bandgap has a substantial frequency bandwidth at the air line. Properties of the first higher order bandgap were investigated rigorously in^8^ with different apex radius curvatures and strut thicknesses. Total bandwidth of the apexes’ fundamental bandgap is reduced dramatically in the triangular structure compared with the square structure. Figure 2 demonstrates that while the apex band diagrams can approximate the lowest normalized frequency of the structures’ fundamental bandgap, they cannot predict accurately the frequency bandwidth of the structures’ bandgaps.

**Figure 3:**
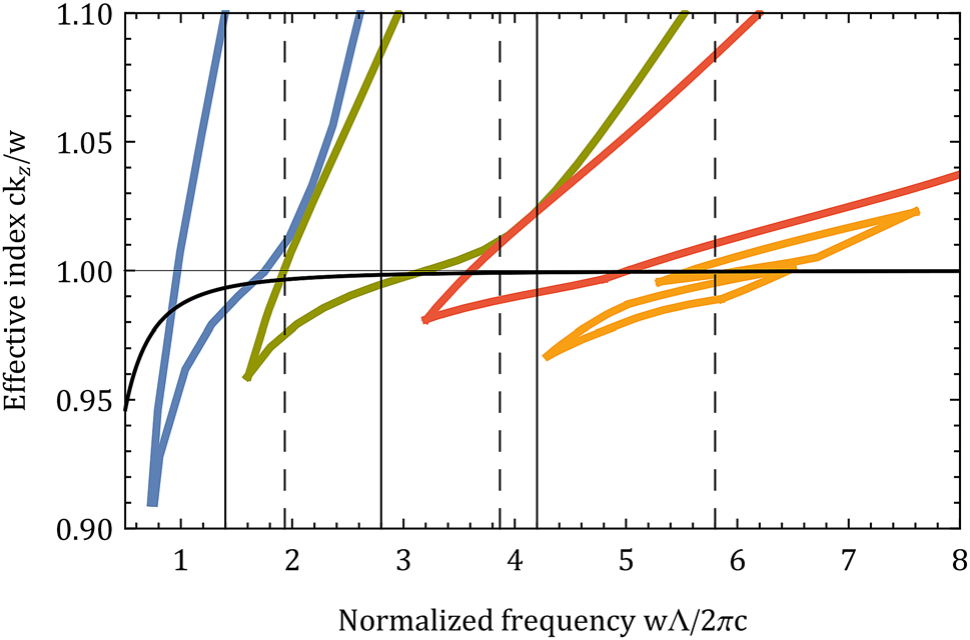
Fundamental bandgaps of the hexagonal (blue), square (green) and triangular (red) structures with an ${0.15\Lambda }$ apex curvature and ${0.01\Lambda }$ strut thickness. The triangular structure has two higher order bandgaps (yellow); yet, only the first higher order bandgap has a substantial frequency bandwidth. The SH and TH could be supported at normalized frequencies of 4.2, 2.8 and 1.4 by combining the properties of the hexagonal, square and triangular structures (black vertical lines). The SH and TH could also be supported at normalized frequencies of 5.8, $\sim 3.87$ and $\sim 1.93$ by combining the properties of the square and triangular structures (black vertical dashed lines). For reference, the FM effective index of a capillary^29^ is shown for wavelength and core diameter of 1500 nm and 14.2 $\upmu \hbox {m}$, respectively.

Band diagrams of the hexagonal, square and triangular structures with an ${0.15\Lambda }$ apex curvature and ${0.01\Lambda }$ strut thickness are shown in Fig. 3. High air-filling structures with a smaller apex separation have a fundamental bandgap at a higher normalized frequency. If somehow several properties of these high air-filling structures could be combined there would be two obvious approaches to guide the SH and TH. The first approach would be to combine the guiding properties of the hexagonal, square and triangular structures. The hexagonal fundamental bandgap would guide the FM, the square and triangular fundamental bandgaps would guide the SH and TH. This approach has two disadvantages: (a) supporting the FM and the first two harmonics requires a structure with three different apexes. Since each apex is expected to reduce the frequency bandwidth of the other apex bandgaps as a result of interference effects, reducing the number of different apexes in the structure is preferred for high harmonic guidance. (b) the FM would be guided at a low normalized frequency, thus would have a high transmission loss in the fundamental bandgap. This high transmission loss could be reduced by increasing the number of periods in the cladding^10^ or by designing a customized core^28^; yet, these methods would make the fabrication of the fiber much more difficult. The second approach would be to combine the guiding properties of the square and triangular properties and guide the FM in the square fundamental bandgap and use the triangular fundamental and first higher order bandgaps to guide the SH and TH. This approach eliminates the disadvantages mentioned previously: (a) the structure would have only two different apexes instead of three, which would reduce apex interference effects and make the fabrication of the fiber easier and (b) the FM would be guided in the fundamental square bandgap at a higher normalized frequency, thus reducing the transmission loss of the FM.

Since the fundamental bandgap is shifted to lower normalized frequencies as the apex separation increases, and since guiding with a low transmission loss is difficult when the fundamental bandgap is at a low normalized frequency, it is preferred to construct hybrid structures that only have hexagonal, square and triangular tilings and avoid more complicated tilings with larger apex separation such as dodecagons. It is also preferred to use tilings that are invariant for ${60}{^{\circ }}$ rotations (such as the p6m symmetry tilings) since they facilitate construction of a circular-shaped core. The square and triangular apexes are located at the center of the square and triangular tilings and therefore, obtaining the guiding properties of the square and triangular structure could be realized in a single structure by simply placing the center of the preform capillaries on the vertices of a hybrid structure with square and triangular tilings. The higher order bandgap of the triangular structure is generated with struts of length ${\Lambda /\sqrt{3}}$, thus enforcing another limitation on the desired hybrid structure.

**Figure 4:**
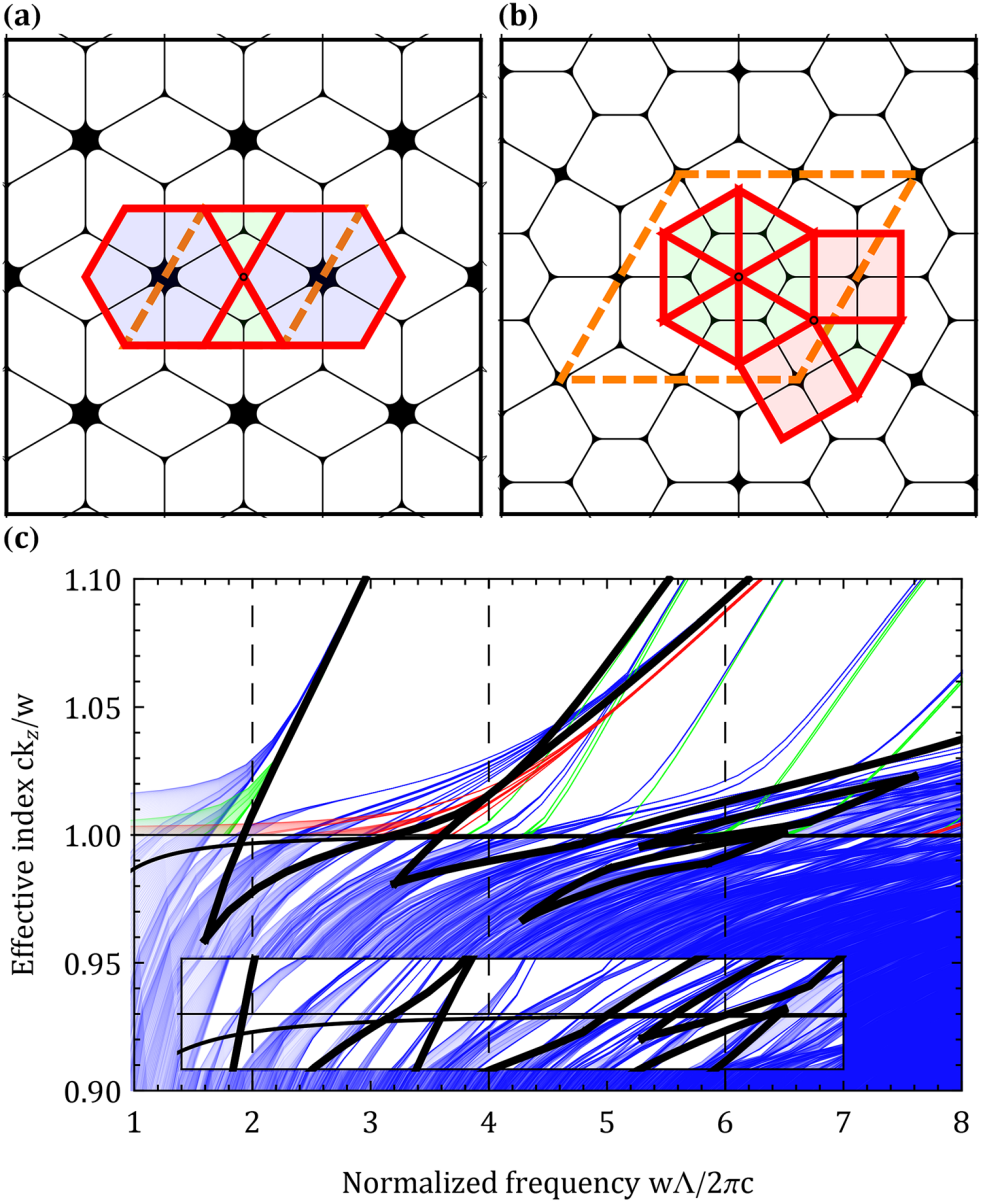
(**a**) Trihexagonal uniform tiling and (**b**) [3^6^;3^2^.4.3.4] 2-uniform tiling, and their corresponding high air-filling structures. (**c**) Band diagram of the [3^6^;3^2^.4.3.4] structure, green and red curves are the band diagrams of the square and triangular apexes, respectively. Bandgaps of the square and triangular structures (see Fig. 3) are shown in black bold. Figure inset shows the structures’ band diagram within the effective index range of $1\pm 0.01$.

Out of all eleven uniform tilings, none have only square and triangular tilings with a p6m symmetry; yet, there may be a uniform tiling that supports the TH. The trihexagonal tiling (Fig. 4a) is a good candidate since the hexagonal fundamental bandgap can guide the FM and the triangular fundamental bandgap can guide the TH. Out of all 2-uniforn tilings^20,30^ the [3^6^;3^2^.4.3.4] tiling (Fig. 4b) fulfills all restrictions mentioned above. The struts in the structure have two different lengths: ${\Lambda /\sqrt{3}}$, and ${\Lambda (1+\sqrt{3})/2\sqrt{3}}$. Struts of length ${\Lambda /\sqrt{3}}$ should support the higher order bandgap of the triangular structure; yet, it is unclear if the struts of length ${\Lambda (1+\sqrt{3})/2\sqrt{3}}$ will prevent the utilization of this higher order bandgap as a result of interference effects. Band diagram of the [3^6^;3^2^.4.3.4] structure with ${0.15\Lambda }$ apex curvature and ${0.01\Lambda }$ strut thickness is shown in Fig. 4c. The [3^6^;3^2^.4.3.4] structure band diagram has promising results for high harmonic guidance. Although there are several triangular apex modes crossing through the square fundamental bandgap, there is still a bandgap near the normalized frequency of ${\sim 2}$. Similarly, several square apex modes cross through the triangular fundamental bandgap; yet they do not completely block it, and there are still several small bandgaps near the SH.

**Figure 5:**
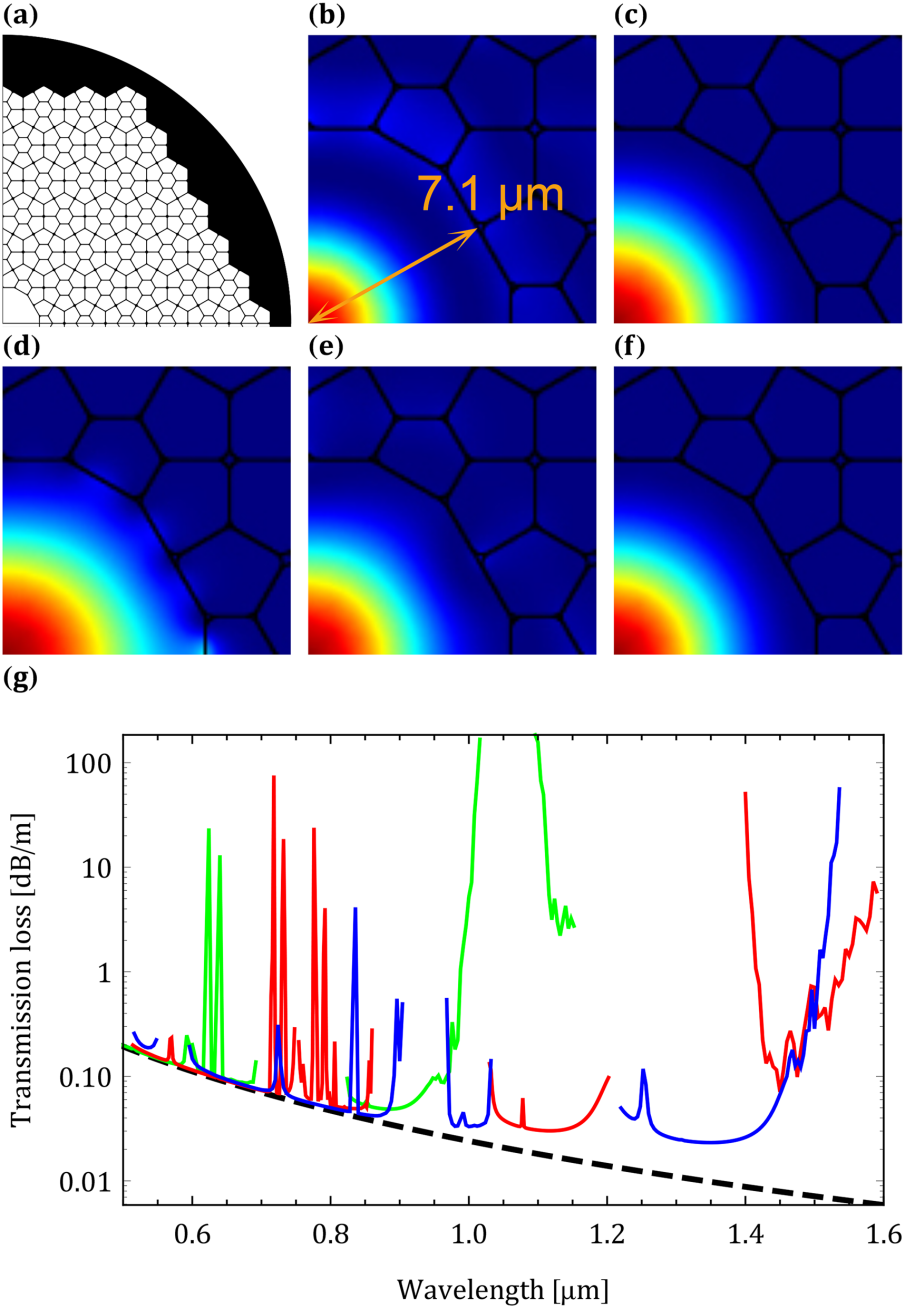
(**a**) [3^6^;3^2^.4.3.4] HC-PCF layout. z component of the Poynting vector of the (**b**) FM, (**c**) SH and (**d**) TH with the [3^6^;3^2^.4.3.4] structure. Mode spatial distributions at (**e**) 1064 nm and (**f**) 800 nm are also shown. (**g**) Scattering loss (black dashed curve) and transmission loss of the [3^6^;3^2^.4.3.4] HC-PCF with apex curvatures of 300 nm (green curve), 450 nm (red curve) and 600 nm (blue curve).

The effects of silica dispersion and the finite cladding were investigated with the finite element method. ${\Lambda ={3} \,{\upmu \hbox {m}}}$ was selected to design the fiber for a 1500 nm fundamental laser source (such as an Er/Yb fiber laser source). Strut thicknesses and apex curvatures were t=30 nm and r=450 nm, respectively. The 19-cell core thickness and curvatures between the dodecagon core edges were identical to the structures strut thickness and apex curvatures. The [3^6^;3^2^.4.3.4] HC-PCF layout is shown in Fig. 5a. With these parameters, guiding the FM, SH and TH is possible at ${\sim 1570} \,\hbox {nm}$, ${\sim 785} \,\hbox {nm}$ and ${\sim 523} \,\hbox {nm}$. Figure 5b–d show the z component of the Poynting vector near the hollow-core at these wavelengths. The spatial overlap between the FM, SH and TH is exceptionally good. Intensity full width at half maximum (FWHM) of the FM, SH and TH is ${\sim 5.3} \,{\upmu \hbox {m}}$, ${\sim 6.7} \,{\upmu \hbox {m}}$ and ${\sim 8.4} \,{\upmu \hbox {m}}$, respectively. Scattering loss in HC-PCFs is known to be proportional to ${\propto \lambda ^{-3}}$^4^ where ${\lambda }$ is the wavelength. Since the lowest transmission loss of a HC-PCF with a core diameter very similar to the [3^6^;3^2^.4.3.4] HC-PCFs’ core diameter is known^31^, the constant of proportionality can be calculated. The reference HC-PCF used for calculating the scattering loss of the [3^6^;3^2^.4.3.4] HC-PCF had the following specifications: lowest loss at 1064 nm was 20 dB/km and core diameter was ${\sim 13} \,{\upmu \hbox {m}}$^31^. Transmission loss of the [3^6^;3^2^.4.3.4] HC-PCF can be approximated by adding the confinement loss and scattering loss (Fig. 5g). Transmission loss at the FM, SH and TH is ${\sim 2.8}\hbox { dB/m}$, ${\sim 0.059}\hbox { dB/m}$ and ${\sim 0.18}\hbox { dB/m}$, respectively. Transmission losses of the [3^6^;3^2^.4.3.4] HC-PCF are also shown in Fig. 5g for apex curvatures of r=300 nm and r=600 nm. By inspecting carefully Fig. 4c it is clear that the high transmission regions centered at ${\sim 1.1} \,{\upmu \hbox {m}}$ and ${\sim 1.45} \,{\upmu \hbox {m}}$ are associated with the square apex fundamental bandgap and that the high transmission region that spans the wavelength range of ${\sim 0.50}$-${0.85} \,{\upmu \hbox {m}}$ is associated with the triangular apex fundamental bandgap. Since there are 245 triangular apexes and only 86 square apexes in a quarter of the fiber (including the apexes on the *x* and *y* axes), confinement from the triangular apexes is much stronger compared with the confinement from the square apexes. In addition, the triangular apex fundamental bandgaps’ central frequency is located at a higher normalized frequency compared with the square apex fundamental bandgaps’ central frequency. Usually, a better transmission loss can be obtained when the fundamental bandgaps’ central frequency is located at a higher normalized frequency^10^. Balance between the scattering loss, number of resonators in the finite cladding and location of the fundamental bandgaps’ central frequency causes the transmission loss to be the lowest at the SH. Low transmission bands, which originate from the resonant character of the 30 nm core strut, are expected above ${9} \,{\upmu \hbox {m}}$ and below 200 nm. For example, low transmission bands for ${N=0.5}$ (see equation (2) in^19^) are centered at ${\sim 9.9} \,{\upmu \hbox {m}}$, ${\sim 124}$ nm and ${\sim 82}$ nm. Discontinuities and oscillations in the transmission loss shown in Fig. 5g originate from: (a) discontinuities in the band diagram at the air line and (b) anti-crossing of the fundamental mode with surface modes.

The 1064 nm (Nd:YAG) and 800 nm (Ti:Sapphire) laser wavelengths are also supported with the [3^6^;3^2^.4.3.4] structure. Fig. 5e,f show the Gaussian mode distributions at these wavelengths. The transmission loss at 1064 nm and 800 nm is ${\sim 0.034}\hbox { dB/m}$ and ${\sim 0.053}\hbox { dB/m}$, respectively. By scaling ${\Lambda }$, small blue and red shifts of all bandgaps are feasible. The struts’ thickness, which is the [3^6^;3^2^.4.3.4] structure’s smallest feature size, limits the blue shift of all bandgaps.

Many n-uniform tilings have been reported^32^, this implies that there may be many more tilings that will allow to guide light at multiple wavelengths with HC-PCFs. For example, the 3-uniform [3^6^;3^3^.4^2^;3^2^.4.3.4] and [3^6^;3^2^.4.3.4;3^2^.4.3.4] tilings have square and triangular apexes and also have struts of length ${\Lambda /\sqrt{3}}$ (with p6m symmetry). As n increases, the n-uniform tilings have a larger unit cell size, thus computing the band diagrams will be much more challenging. For example, the rhombus unit cell of the [3^6^;3^2^.4.3.4] 2-uniform tiling (Fig. 4b) has an edge length of ${\Lambda (1+\sqrt{3})}$; yet, the [3^6^;3^3^.4^2^;3^2^.4.3.4] and [3^6^;3^2^.4.3.4;3^2^.4.3.4] 3-uniform tilings have a larger edge length of ${\Lambda (2+\sqrt{3})}$. Tiling and pattern theory is linked to the stack and draw fabrication method^23,33^; therefore, fabrication of the [3^6^;3^2^.4.3.4] structure could be realized if the preform capillaries were somehow centered on the 2-uniform tiling vertices. One method to center capillaries on the vertices of the [3^6^;3^2^.4.3.4] 2-uniform tiling was reported in^34^. By stacking A7 unit cells in the preform, a solid core Archimedean-like PCF was fabricated. In this report the authors neglected to point out that the Archimedean-like structure is actually the [3^6^;3^2^.4.3.4] structure. Stacking A7 unit cells may be a promising method for fabricating HC-PCFs that contain the [3^6^;3^2^.4.3.4] structure. Another method to fabricate the [3^6^;3^2^.4.3.4] tiling is by preparing a PCF preform with ultrasonic drilling and estimating the air hole geometry change ratio with the capillary Navier-Stokes model^35^. The final air hole diameter according to this model is expressed with the following equations^36^:

1 $$\begin{aligned}{} &h_{1}=\exp {\left( -\dfrac{\beta }{2}-P\exp {(-\beta )}\right) }\left( h_{10}\exp {(P)}- G\int _{0}^{L}\exp {\left( -\dfrac{\beta u}{2L}+P\exp {\left( -\dfrac{\beta u}{L}\right) }\right) }\mathrm {d}u\right) \\&G=\dfrac{\gamma }{2\mu W_{f}} \qquad P=\dfrac{pL}{2\beta \mu W_{f}} \qquad \beta =\ln {\left( \dfrac{W_{d}}{W_{f}}\right) } \qquad h_{2}=h_{20}\exp {\left( -\dfrac{\beta }{2}\right) } \end{aligned}$$

where ${h_{10}}$ is the initial air hole diameter, ${h_{20}}$ is the initial fiber outer diameter, ${h_2}$ is the final fiber outer diameter, ${\gamma }$ is the surface tension, ${\mu }$ is the viscosity, *p* is the hole overpressure, ${W_f}$ and ${W_d}$ are the feed and draw speeds and *L* is the heating zone length. The final air hole diameter can be expressed more elegantly with the imaginary error function:

2 $$\begin{aligned} h_{1}=\exp {\left( -\dfrac{\beta }{2}-P\exp {(-\beta )}\right) }\left( h_{10}\exp {(P)} -\dfrac{\sqrt{\pi }GL}{\sqrt{P}\beta }\left( {{\,\mathrm{erfi}\,}}{\left( \sqrt{P}\right) } -{{\,\mathrm{erfi}\,}}{\left( \sqrt{P}\exp {\left( -\dfrac{\beta }{2}\right) }\right) }\right) \right) \end{aligned}$$

Using Eq. (2), the geometry change ratio is expressed with the following equation:

3 $$\begin{aligned} C=\dfrac{h_{1}h_{20}}{h_{2}h_{10}}=\dfrac{\exp {\left( -P\exp {(-\beta )} \right) }}{h_{10}}\left( h_{10}\exp {(P)}-\dfrac{\sqrt{\pi }GL}{\sqrt{P}\beta } \left( {{\,\mathrm{erfi}\,}}{\left( \sqrt{P}\right) }-{{\,\mathrm{erfi}\,}}{\left( \sqrt{P} \exp {\left( -\dfrac{\beta }{2}\right) }\right) }\right) \right) \end{aligned}$$

**Figure 6:**
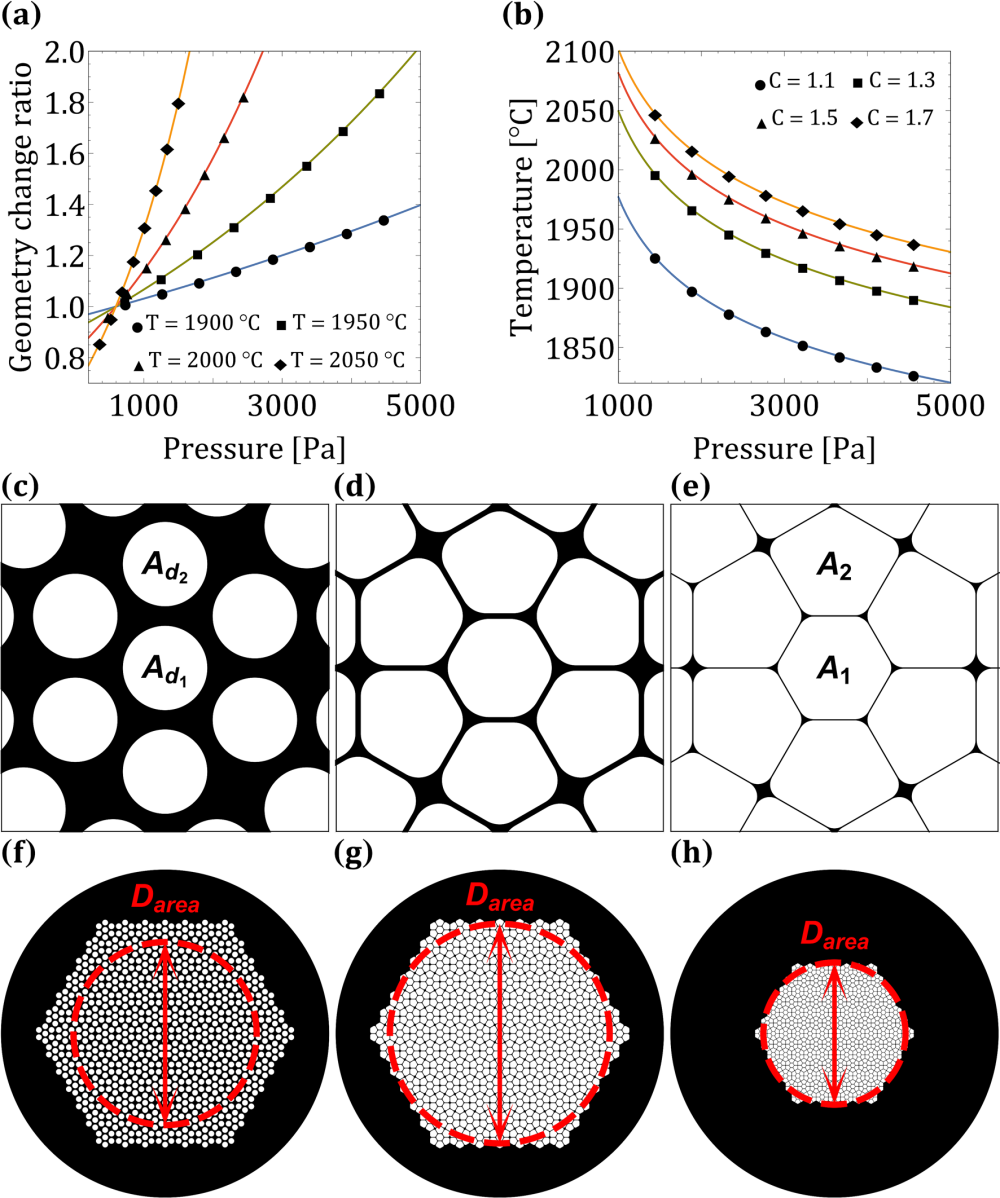
(**a**,**b**) Geometry change ratio for different pressures and temperatures. (**c**,**f**) Preform, (**d**,**g**) cane and (**e**,**h**) [3^6^;3^2^.4.3.4] PCF calculated with the Navier-Stokes model. The cane drawn in the first stage (**g**) was inserted into a sleeve that had an 11 mm outer diameter. Cane and sleeve were drawn in the second stage to the [3^6^;3^2^.4.3.4] PCF.

Air hole expansion requires that the geometry change ratio fulfill the condition ${C>1}$. In^35^ the air hole diameter was defined as the diameter of a circle that has the same circumference as the total of all the inner hole perimeters ${D_{perimeter}}$. This diameter definition had a better correlation with experimental results compared with other air hole diameter definitions, such as the diameter of a circle that has the same area as the total area of the inner holes ${D_{area}}$. In the extended work of^35^ (Roman Kostecki’s thesis), he showed that the former and latter definitions have a good correlation with experimental results, with ${D_{perimeter}}$ giving slightly better results. The equations for the perimeters of the two different air holes in the [3^6^;3^2^.4.3.4] tiling are (see Fig. 6e):

4 $$\begin{aligned} S_{1}= {} 2\left( r_{1}\left( \pi -2\sqrt{3}\right) +\sqrt{3} \left( \Lambda -t_{1}\right) \right) \end{aligned}$$

5 $$\begin{aligned} S_{2}= \dfrac{1}{2}\left( 2\left( r_{2}\left( \pi -2\sqrt{3}\right) +\sqrt{3}\left( \Lambda -t_{2}\right) \right) +2\left( r_{2}\left( \pi -4\right) +2\left( \Lambda -t_{2}\right) \right) \right) \end{aligned}$$

Where *r* is the apex curvature and *t* is the strut thickness. When dealing with high air-filling PCFs, defining the air hole diameter as ${D_{perimeter}}$ can lead to unphysical results. Assuming the [3^6^;3^2^.4.3.4] tiling spans the hollow-core region, there are 127 ${S_1}$ and 762 ${S_2}$ air hole perimeters in the PCF. ${D_{perimeter}}$ of all the air holes is ${\sim 2963.8} \,{\upmu \hbox {m}}$, which is much larger than the air hole region, and is likely to be larger than any desired final outer diameter ${h_2}$. For this reason ${D_{area}}$ is much more suitable for modeling PCFs’ air hole geometry change ratio. The air hole region diameter ${D_{area}}$ of the [3^6^;3^2^.4.3.4] PCF is shown in Fig. 6h and is equal to ${\sim 95.4} \,{\upmu \hbox {m}}$. The equations for the area of the two different air holes in the [3^6^;3^2^.4.3.4] tiling are (see Fig. 6e):

6 $$\begin{aligned} A_{1}= {} \dfrac{\sqrt{3}}{2}\left( \Lambda -t_{1}\right) ^2+r_{1}^2 \left( \pi -2\sqrt{3}\right) \end{aligned}$$

7 $$\begin{aligned} A_{2}= {} \dfrac{1}{2}\left( \dfrac{\sqrt{3}}{2}\left( \Lambda -t_{2}\right) ^2 +r_{2}^2\left( \pi -2\sqrt{3}\right) +\left( \Lambda -t_{2}\right) ^2+r_{2}^2 \left( \pi -4\right) \right) \end{aligned}$$

If we rewrite the geometry change ratio in terms of the air hole area we obtain:

8 $$\begin{aligned} C=\dfrac{h_{1}h_{20}}{h_{2}h_{10}}=\dfrac{h_{20}\sqrt{A}}{h_{2}\sqrt{A_{10}}} \quad \longrightarrow \quad A=A_{10}\left( \dfrac{h_{2}}{h_{20}}\right) ^2C^2 \end{aligned}$$

Where *A* is the total final air hole area and ${A_{10}}$ is the total initial air hole area. Since ${A_{10}}$ may be constructed of different air holes in the preform, *A* can be expressed with the following equation:

9 $$\begin{aligned} A=127A_{1}+762A_{2}=A_{10}\left( \dfrac{h_{2}}{h_{20}}\right) ^2C^2 =\left( 127A_{d_1}+762A_{d_2}\right) \left( \dfrac{h_{2}}{h_{20}}\right) ^2C^2 \end{aligned}$$

Where ${A_{d_1}}$ and ${A_{d_2}}$ are the initial preform air holes with diameters ${d_1}$ and ${d_2}$, respectively. Assuming the preform air holes ${A_{d_1}}$ and ${A_{d_2}}$ change their area independently, they will transition according to *C* into final air holes ${A_1}$ and ${A_2}$ that are expressed with the following equations:

10 $$\begin{aligned} A_1= {} A_{d_1}\left( \dfrac{h_{2}}{h_{20}}\right) ^2C^2 \end{aligned}$$

11 $$\begin{aligned} A_2= {} A_{d_2}\left( \dfrac{h_{2}}{h_{20}}\right) ^2C^2 \end{aligned}$$

According to equations (10) and (11) obtaining an identical *r*, *t* and ${\Lambda }$ in the final PCF is only possible if the ratio between the final air hole areas is equal to the square of the ratio between ${d_1}$ and ${d_2}$:

12 $$\begin{aligned} \dfrac{A_1}{A_2}=\dfrac{A_{d_1}}{A_{d_2}}=\left( \dfrac{d_1}{d_2}\right) ^2 \end{aligned}$$

For the [3^6^;3^2^.4.3.4] PCF shown in Fig. 6e with parameters: ${r=0.15\Lambda }$, ${t=0.01\Lambda }$ and ${\Lambda ={3} \,{\upmu \hbox {m}}}$ we obtain ${d_2/d_1\sim 1.035}$. As shown in^35^, the Navier-Stokes model will not work properly without applying pressure and temperature offsets ${p_\epsilon }$ and ${T_\epsilon }$. Figure 6a,b show the calculated geometry change ratio for different pressures and temperatures (equation (3)). The following parameters were used in these calculations: ${p_\epsilon ={-479.9}{\hbox { Pa}}}$, ${T_\epsilon ={-147.5}^{\circ }{\hbox {C}}}$, ${W_f={1.5}\hbox { mm/min}}$, ${\beta \sim 8.63}$, ${L={0.039}\hbox { m}}$, ${\gamma ={0.3}\hbox { N/m}}$^37^, ${h_{10}\sim {0.005}\hbox { m}}$. Glass viscosity was calculated with the following equation^38^:

13 $$\begin{aligned} \mu =5.8\times 10^{-8}\exp {\left( \dfrac{ {515400}{\mathrm{J/mol}}}{RT}\right) }~\hbox {[Pa s]} \end{aligned}$$

Where *R* is the gas constant in units of J/K mol and *T* is the temperature in units of K. Even though equation (3) can predict the geometry change ratio of the PCF, it cannot predict the final apex curvature, strut thickness and center air hole spacing. For a specified *C*, ${h_{20}}$, ${h_2}$ and preform air hole diameters ${d_1}$ and ${d_2}$, there are many apex curvatures *r*, strut thicknesses *t* and center air hole spacing ${\Lambda }$ that have the same final air hole areas ${A_1}$ and ${A_2}$. Therefore, finding the exact *r*, *t* and ${\Lambda }$ for a specified *C*, ${h_{20}}$, ${h_2}$ and preform air hole diameters ${d_1}$ and ${d_2}$ must be done experimentally. Figure 6a,b show different combination of pressures and temperatures that have an identical *C*, each combination will have the same air hole area; yet, *r*, *t* and ${\Lambda }$ will be different. With equations (3), (6), (7), (10), (11) and (12) it is possible to estimate the PCF layout during the fiber draw. The Navier-Stokes model is not limited to a single stage fabrication process. A similar [3^6^;3^2^.4.3.4] PCF to the one shown in Fig. 5a can be realized with a two stage cane and sleeve process^39^. Figure 6f–h show the cross-section of the [3^6^;3^2^.4.3.4] PCF during the fiber draw calculated with the Navier-Stokes model. In the first stage, the preform (Fig. 6f) was drawn to a cane (Fig. 6g). This cane was inserted into a sleeve that had a 11 mm outer diameter. In the second stage, the cane and sleeve were drawn to the [3^6^;3^2^.4.3.4] PCF. In the first cane stage, the draw parameters were: ${C=1.2}$, ${d_1={2}\hbox { mm}}$, ${d_2\sim {2.07}\hbox { mm}}$, ${\Lambda _{preform}\sim {2.45}\hbox { mm}}$, ${h_{20}={110}\hbox { mm}}$, ${h_{2}={4}\hbox { mm}}$. Cane curvatures were ${r_1=0.3\Lambda _{cane}}$ and ${r_2\sim 0.247\Lambda _{cane}}$, cane strut thicknesses were ${t=0.05\Lambda _{cane}}$ and the cane air hole spacing was ${\Lambda _{cane}\sim {89.16} \,{\upmu \hbox {m}}}$. In the second sleeve stage, the draw parameters were: ${C\sim 1.78}$, ${h_{20}={11}\hbox { mm}}$, ${h_{2}={220} \,{\upmu \hbox {m}}}$. Final [3^6^;3^2^.4.3.4] PCF has the same curvatures, strut thicknesses and air hole spacing as the [3^6^;3^2^.4.3.4] HC-PCF shown in Fig. 5a. Figure 6 demonstrates that the Navier-Stokes model can help estimate the geometry change ratio of high air-filling PCFs during the stack and draw fabrication process.

## Conclusions

In conclusion, we demonstrated how to apply tiling and pattern theory in the design of HC-PCFs for SH and TH guidance. Since the apexes of the hexagonal, square and triangular structures are located at the center of their tilings, it is possible to obtain a single cladding structure with multiple spectral bandgaps by placing capillaries on the vertices of uniform or n-uniform tilings. Since the bandgap properties of high air-filling structures are mostly caused by the apexes, combining different apexes in a single structure can lead to claddings that support the FM, SH and TH. Not only does the [3^6^;3^2^.4.3.4] structure support the SH and TH with a ${\sim 1570}$ nm fundamental laser source, it can also guide the wavelengths of Ti:Sapphire and Nd:YAG laser sources with low transmission losses.

## Methods

Band diagrams were computed with the plane wave expansion method. The number of plane waves in these calculations was 2791. Finite element calculations were performed with COMSOL. The number of elements in these calculations was 3373305 and the perfectly matched layer (PML) extended ${10} \,{\upmu \hbox {m}}$ in the radial direction. Material dispersion was included with a three-term Sellmeier equation, absorption losses were included by adding to the silica regions a constant ${10^{-7}}$ imaginary refractive index^40^.

## Data Availability

All data generated or analysed during this study are included in this published article (and its Supplementary Information files).
